# Mental health and addiction concerns in northern Ontario: A cross-sectional study of sociodemographic predictors, access barriers, and service needs

**DOI:** 10.1371/journal.pmen.0000503

**Published:** 2025-12-04

**Authors:** Michelle Hirsch, Anthony Levitt, Roula Markoulakis

**Affiliations:** 1 Sunnybrook Research Institute, Toronto, Ontario, Canada; 2 Department of Psychology, York University, Toronto, Ontario, Canada; 3 Department of Psychiatry, University of Toronto, Toronto, Ontario, Canada; 4 Department of Psychiatry, Sunnybrook Health Sciences Centre, Toronto, Ontario, Canada; 5 Department of Occupational Science and Occupational Therapy, University of Toronto, Toronto, Ontario, Canada; PLOS: Public Library of Science, UNITED KINGDOM OF GREAT BRITAIN AND NORTHERN IRELAND

## Abstract

Access to mental health and/or addiction (MHA) services is limited in rural and remote regions, especially in geographically diverse areas such as Ontario, Canada. Moreover, available services may not be able to address the unique needs of those seeking support. To effectively address local MHA service needs, it is necessary to understand predictors of MHA concerns and the experiences of those accessing care in rural areas such as northern Ontario. The current study focused on individuals living in northern Ontario and aimed to 1. Identify sociodemographic factors that predict their MHA concerns; 2. Identify common barriers experienced by those seeking MHA services; and 3. Explore their MHA support needs. Survey data were collected online from 500 northern Ontario residents (aged 18+) between January and March 2022. Univariate statistics were used to describe MHA service access, barriers, and needs, and adjusted multiple logistic regression was conducted to assess predictors of MHA concerns. Younger age (Odds Ratio (OR) = 0.972), low socioeconomic status (middle: OR = 0.491; high: OR = 0.436), identifying as non-straight/non-heterosexual (straight/heterosexual: OR = 0.336), identifying as married (unmarried: OR = 0.507), and dissatisfaction with social support (OR = 6.410) were significant predictors of MHA concerns. Although most (76.8%) of the sample reported MHA concerns, less than a quarter of the sample accessed support. Most frequently accessed services tended to be less specialized, and most frequently reported access barriers were mainly systemic. The current study describes predictors of MHA concern as well as the unique MHA-service-related experiences and needs of northern Ontario residents. These findings may be considered in efforts to develop MHA tools and supports that align with local needs.

## 1. Introduction

Within Canada, experience of mental health and/or addiction (MHA) problems is not uncommon [1]. In fact, the prevalence of these problems, in Canada and beyond, appear to be increasing over recent years [2,3]. Accordingly, it is critical to understand potential risk factors for experiencing MHA concerns. Existing literature highlights geographical location as a prominent sociodemographic predictor of MHA concerns. In comparing different regions within Ontario, for example, higher rates of mental health (e.g., depression [4]) and substance (e.g., alcohol) use are found in urban and rural northern Ontario locales relative to other regions [5,6]. Higher rates of self-harm hospitalizations are also observed in more rural compared to urban regions [7], potentially signalling higher rates of suicidality. Prior qualitative work conducted in northern Ontario identified that participants who were women, seniors, 2SLGBTQIA + , Indigenous, homeless, lower income, and unattached often described difficulties associated with their mental health and/or substance use [8]. Although such exploratory work has been important for developing initial understandings of the relationship between MHA needs and sociodemographics in rural/remote regions, among these groups, even less is known about MHA service use patterns and preferences.

Within Canada and across other geographically diverse countries, rural and remote communities experience mental health care inequities [9,10]. Individuals seeking MHA support in such regions encounter unique barriers to obtaining appropriate care. For example, residents of rural British Columbia when asked to list location-related barriers to accessing healthcare services, included some that involved making safety tradeoffs (i.e., travelling during poor outdoor conditions), healthcare delivery efficiency, and out-of-pocket expenses associated with travel [11]. In Nova Scotia, community-level barriers to accessing mental health services include lack of privacy due to small community size, few available services and lack of cultural diversity within available services, and inconvenient service location with associated travel costs [12]. Thus, despite the vast prevalence of MHA concerns within rural regions, an imbalance exists between the needs, preferences, and accessibility of services available to residents.

Northern Ontario, specifically, comprises rural and remote communities facing many barriers to MHA service access [13], including major cities such as Greater Sudbury, Thunder Bay, Sault Ste. Marie, North Bay, and Timmins. Like other rural regions, northern Ontario has limited public transportation options [14], fewer physicians, and fewer pharmacists compared to other Ontario locales [15]. While those living in southern Ontario can typically reach hospitals in about 30 minutes (by car, walking, or a mixture), northern Ontario residents cannot reach the nearest hospital within the same time frame, regardless of how they travel there [16]. The wide geographic dispersions of healthcare services within northern Ontario sometimes require residents to travel to southern regions to obtain mental health support [17]. Families in this region similarly experience greater difficulty accessing MHA services for their youth [18], with stigma, lack of anonymity [19], financial difficulties, lack of information and resources, and far distances to access services among the barriers experienced [20]. Limited coordination of services and poor follow-up have also been reported as obstacles to service access [8]. In light of such barriers to MHA care access, high rates of hospitalizations and emergency department (ED) visits for MHA concerns have been observed in northern Ontario [8,21]. Such observations may, in part, be driven by prevalent opioid use found in northern Ontario specifically [22]. Increasing access to consistent and specialized MHA healthcare and community settings has been suggested as an approach to reducing ED utilization [8]. These findings illuminate the vast barriers to service access that Northern Ontario residents face.

Additional MHA concerns and access barriers might ensue due to the diverse sociodemographic profiles of individuals living in northern Ontario. Compared to southern Ontario, individuals in northern Ontario with concurrent disorders are more impoverished and less educated [23]. Areas with lower socioeconomic status (SES) have a higher likelihood of unmet mental health needs [13]. Moreover, approximately 18% of the northern Ontario population are Indigenous Peoples, a stark contrast to the 2.4% Indigenous population in Ontario overall [24]. Indigenous post-secondary students, compared to non-Indigenous students, show higher probabilities of self-harm, suicidal ideation, and suicide attempts [25]. Indigenous students are also more likely to self-report substance use, have a higher probability of mood and anxiety disorder diagnosis, and, overall, have higher rates of MHA concerns compared to non-Indigenous counterparts [25]. In view of these sociodemographic factors that may impact the prevalence of MHA concerns and therefore well-being and quality of life of a relatively large number of residents of northern Ontario, a lack of access to MHA services [10] is particularly concerning.

Many of the previous studies on MHA concern experiences within northern Ontario have been qualitative in nature. In addition, there are few recent studies investigating factors like satisfaction with available services and perceptions of barriers to service access within northern Ontario, specifically. The current study addresses these gaps by employing a cross-sectional, quantitative approach, using a subset of the survey data collected for the COVID-19 Mental Health & Addictions Service Impacts & Care Needs (MASC) study [26]. This study investigates a community sample of residents of northern Ontario to: 1. Identify sociodemographic factors that predict their MHA concerns; 2. Identify common barriers experienced by those seeking MHA services; and 3. Explore their MHA support preferences and needs. Adjusted logistic regression and descriptive analyses are used to accomplish these aims, respectively, and results are contextualized alongside their implications for improved MHA support in northern Ontario.

## 2. Methods

### 2.1. Ethics statement

The authors assert that all procedures contributing to the current study comply with: 1. Ethical standards of relevant national and institutional committees on an experimentation; 2. The Helsinki Declaration of 1975 (as revised in 2008).

### 2.1. Study design and participants

The present study aimed to investigate sociodemographic variables that may correlate with screening for MHA concerns. Furthermore, we aimed to understand the experiences and needs of those seeking MHA services. To accomplish this, the current study used phase 3 survey data collected (between January and March 2022) as part of the MASC study [26]. The study collected information on participants’ sociodemographic characteristics and MHA experiences and perspectives. Non-probabilistic sampling was used in that only individuals registered with Delvinia’s AskingCanadians [27] were randomly sent the survey link if their profiles matched a priori quotas. During the sampling process, targeted oversampling was conducted for northern Ontario, given that this region is less densely populated. The pool from which participants were sampled was benchmarked by region, age, and gender to ensure provincial representativeness. See the study protocol for additional information on participant inclusion/exclusion criteria, participant recruitment, and sampling methods [26]. Participants aged 18 years or older living in northeastern and northwestern (northern) Ontario, Canada, were included in the current analysis (*n* = 500), and this data was accessed on June 3, 2024. During and following data collection, the authors did not have access to information that could identify individual participants. All study procedures were approved by Sunnybrook Health Sciences Research Ethics Board (ref. 1931), and written informed consent was obtained from study participants.

### 2.2. Variables and measures

As part of the COVID-19 MASC study, respondents’ sociodemographic backgrounds, MHA concerns, and experiences with MHA services (i.e., access barriers, support needs/preferences, etc.) were collected from participants residing across Ontario using several measures [26]. The following describes the subset of measures that were utilized in the current study.

Participants indicated their age, gender, sexual orientation, race, geographical location, highest education level, marital status, and living situation. SES was measured using the MacArthur Scale of Subjective Social Status-Adult Version [28]. Based on tertiles, low SES was categorized to include responses between 1 and 5 (where 1 = worst off), middle SES between 6 and 8, and high SES between 9 and 10 (where 10 = best off). Screening for mental health domain concerns (depression, anxiety, anger, mania, somatic symptoms, suicidal ideation, psychosis, repetitive thoughts and behaviours, dissociation, personality functioning) was measured using the American Psychiatric Association’s Diagnostic Statistical Manual-5 (DSM-5) Self-Related Level 1 Cross-Cutting Symptom Measure, Adult Version [29]. A rating of mild or greater (5-point scale where 0 = none or not at all; 1 = slight or rare, less than a day or two; 2 = mild or several days; 3 = moderate or more than half the days; and 4 = severe or nearly every day) on items within any of the domains signals the potential need for professional assessment. For suicidal ideation and psychosis, a rating of slight or greater signals for a professional assessment need. Substance use was measured using the WHO Alcohol, Smoking and Substance Involvement Screening Test V.3.0 [30], which classifies respondents into either low, moderate, or high risk for substance use and dependence (cannabis, opioids, alcohol, other substances).

Using original materials created for the study [26], participants were asked about several MHA service experiences: services accessed, desired but not accessed, whether seeking new service(s), satisfaction with services/supports received, perceived barriers to service access, perceived helpfulness of certain service types in finding MHA support, preferred mode of access to desired services. Participants indicated (via checklist) the services they accessed and desired but not accessed (e.g., sessions with a private therapist/counsellor, assessment and/or treatment by a psychiatrist, etc.). Participants also indicated whether they were seeking additional services (yes/no response) and their level of satisfaction with accessed services (via a seven-point bipolar Likert scale, where 1 = extremely satisfied and 7 = extremely dissatisfied).

Perceived barriers to service access (e.g., cost too much, unavailable in the preferred format, etc.) were measured using a checklist developed for this study. Using adapted items developed by research team members [31], perceived helpfulness of service types in finding MHA support (e.g., case manager, peer worker, primary healthcare provider, etc.) and preferences for service delivery mode (i.e., variations of in-person, virtual, one-on-one, and group options) were measured (using a five-point Likert scale, where 1 = extremely helpful and 5 = not at all helpful). Finally, a seven-point bipolar Likert scale developed for this study (1 = extremely satisfied, 4 = neutral, 7 = extremely dissatisfied) was used to gauge respondents’ satisfaction with the availability of social supports (e.g., friends, family, romantic partners, etc.). Participants were then asked to identify whether they were the primary caregiver to a child by indicating their number of children.

### 2.3. Statistical analysis

Statistical analysis was conducted using RStudio (version 2024.04.1.748) [32] with a statistical significance threshold set at α = .05. Descriptive statistics were calculated for all sociodemographic, MHA-related, and other-oriented variables of interest: respondents’ age, gender, sexual orientation, SES, race, highest level of education, marital status, living situation, satisfaction with social supports, mental health screening, substance use risk levels, and service experiences. The number of participants with mental health and/or addiction concerns, mental health concerns only, addiction concerns only, and mental health and addiction concerns were also computed.

Adjusted binomial logistic regression was conducted to assess sociodemographic predictors of MHA concerns in the study sample. The predictor variables included in the model – age (continuous), gender, race, sexual orientation, SES, highest level of education, marital status, and satisfaction with social supports – were selected a priori and theoretically informed. The outcome variable, MHA concern, was determined as follows: those meeting a particular threshold (described above) for further clinical inquiry on any domain of the DSM-5 measure used in the current study [29] were considered to have a mental health concern. In contrast, those responding below the threshold on all items were not considered to have a mental health concern. Moreover, those categorized as moderate and/or high risk for substance use and dependence (on any of cannabis, opioids, alcohol, other substances, substances by injection) according to the ASSIST [30] were considered to have an addiction concern. Those with no and/or low risk on all substances were not considered to have an addiction concern. Thus, if participants were considered as having a concern for mental health or addiction only, or mental health and addiction, they were classified in the MHA concern group/outcome variable. Finally, descriptive analysis was used to assess the MHA service-related experiences and needs of respondents.

## 3. Results

### 3.1. Participant characteristics

Sociodemographic information and characteristics of participants (*n* = 500) are presented in Table 1. Approximately half of our sample identified as cis women (*n* = 254; 50.8%), with participant ages ranging from 18 to 92 years (*M *= 48.45, *SD* = 17.12). Participants predominantly identified as heterosexual (*n* = 411; 82.2%) and white (87.8%). Moreover, respondents typically reported having some post-secondary or more (*n* = 385; 77%) education, middle (*n* = 204; 40.8%) SES, and living with others (*n* = 386; 77.2%). Most of the participants were married/common-law (*n* = 294; 58.8%), neutral to dissatisfied (*n* = 253; 50.6%) with social supports, and did not identify as a primary caregiver for at least one child (73%).

**Table 1:** Participant demographics.

	N = 500
	n (%)
**Gender**	
Cis woman	254 (50.8)
Cis man	239 (47.8)
Non-binary	7 (1.4)
**Age**	
18-34	125 (25)
35-44	85 (17)
45-54	100 (20)
55-64	87 (17.4)
65+	103 (20.6)
**Sexual Orientation**	
Straight/Heterosexual	411 (82.2)
2SLGBTQIA+	76 (15.2)
No response	13 (2.6)
**Race**	
Asian (East, South, South-East)	9 (1.8)
Black (African, North American, Caribbean)	8 (1.6)
First Nations, Indigenous, Aboriginal, Metis	17 (3.4)
Latin American	5 (1.0)
Middle Eastern	1 (0.2)
White (European, North American)	439 (87.8)
Mixed Race	17 (3.4)
Other	3 (0.6)
Prefer not to answer	1 (0.2)
**Highest education level**	
High school or less	115 (23)
Some post-secondary or more	385 (77)
**Marital status**	
Married/common-law	294 (58.8)
Unmarried	206 (41.2)
**Living Situation**	
Alone	114 (22.8)
With others	386 (77.2)
**Socioeconomic status**	
Low	191 (38.2)
Middle	204 (40.8)
High	105 (21)
**Satisfaction with social supports**	
Satisfied	247 (49.4)
Neutral	153 (30.6)
Dissatisfied	100 (20)

### 3.2. Predictors of MHA concerns

Adjusted binomial logistic regression was used to assess for sociodemographic characteristics (predictor variables) that might predict MHA concern (outcome variable) in our sample (Table 2). Within the model, no multicollinearity was detected amongst any of the independent predictors (variance inflation factor values < 2.5). Of all predictors included in the model, younger age (OR = 0.972, CI (95%) = 0.957, 1.014, *p* < .001), low SES (middle: OR = 0.491, CI (95%) = 0.272, 0.867, *p* < .05; high: OR = 0.436, CI (95%) = 0.222, 0.847, *p* < .05), 2SLGBTQIA+ sexual orientation (straight/heterosexual: OR = 0.336, CI (95%) = 0.138, 0.731, *p* = .01), identifying as married (unmarried: OR = 0.507, CI (95%) = 0.308, 0.830, *p* < .01), and dissatisfaction with social support(s) (OR = 6.410, CI (95%) = 2.659, 19.144, *p* < .001) were significant predictors of screening for any MHA concern.

**Table 2:** Adjusted logistic regression for predictors of MHA concern.

Predictor	B	Std. Error	OR	CI (95%)	*z*-value	Sig.
Gender (Cis man)	-0.393	0.236	0.675	0.424-1.069	-1.667	0.100
Age	-0.029	0.008	0.972	0.957-1.014	-3.802	<0.001^a^
Education (High school or less)	0.180	0.297	1.197	0.676-2.173	0.605	0.545
SES (Middle)	-0.712	0.294	0.491	0.272-0.867	-2.419	0.016^a^
SES(High)	-0.830	0.340	0.436	0.222-0.847	-2.435	0.015^a^
Sexual orientation (Straight/heterosexual)	-1.089	0.420	0.336	0.138-0.731	-2.591	0.010^a^
Race (Non-White)	0.554	0.476	1.740	0.727-4.850	1.164	0.245
Marital status (Unmarried)	-0.678	0.252	0.507	0.308-0.830	-2.687	0.007^a^
Satisfaction with social support(s) (Neutral)	-0.179	0.255	0.836	0.508-1.383	-0.701	0.483
Satisfaction with social support(s) (Dissatisfied)	1.858	0.494	6.410	2.660-19.144	3.762	<0.001^a^

Abbreviation: OR = odds ratio; CI = confidence interval for OR.

^a^*p* < .05.

### 3.3. MHA service-related experiences

Descriptive statistics for service access based on concern type are presented in Table 3. Of those with MHA concern(s), 76.8% did not access any MHA service.

**Table 3:** Service access by varying concern type.

Concern type	Number of individuals with concern	Accessed service(s)	Did not access service(s)
Mental health and/or addiction	384 (76.8%)	89 (23.2%)	295 (76.8%)
Mental health and addiction	151 (30.2%)	61 (40.4%)	90 (59.6%)
Mental health only	204 (40.8%)	25 (12.3%)	179 (87.7%)
Addiction only	29 (5.80%)	3 (10.3%)	26 (89.7%)

Note. Reported as n (%).

Participants’ MHA service-related experiences are presented in Table 4. Responses to questions concerning MHA services received indicated that most of our sample did not receive any MHA services (*n* = 405, 81%), and 77% of those with MHA concerns, specifically, did not access services (*n* = 295). Across the entire sample, sessions with a private therapist/counsellor (*n* = 49, 9.8%), mental health treatment by a family physician or walk-in doctor (*n* = 35, 7%), and mental health support with a focus on education/employment (*n* = 23, 4.6%) were the top three accessed services. Live-in (*n* = 5, 1%), in-patient hospital (*n* = 2, 0.4%), and “other/unspecified” MHA treatments (*n* = 3, 0.6%) were amongst the lowest accessed services. Participants tended to be more satisfied than dissatisfied with services but were more dissatisfied than satisfied with assessment by a psychiatrist (satisfied: *n* = 3, 37.5%; dissatisfied: *n* = 5, 62.5%), live-in (satisfied: *n* = 1, 20%; dissatisfied: *n* = 4, 80%), and “other/unspecified” treatments (satisfied: *n* = 1, 33.33%; dissatisfied: *n* = 2, 66.67%).

**Table 4:** Respondent MHA service experiences and attitudes.

	n (%)		
**Service type received** ^a^			
None	405 (81)		
Sessions with private therapist/counsellor	49 (9.8)		
Mental health treatment by family physician or walk-in doctor	35 (7)		
Mental health supports with focus on education/employment goals	23 (4.6)		
Treatment by a psychiatrist	15 (3)		
Supports for caregivers of someone with MHA concern	9 (1.8)		
Crisis supports/intervention	9 (1.8)		
Addiction assessment and/or treatment	8 (1.6)		
Assessment by a psychiatrist	8 (1.6)		
Culturally specific mental health services	6 (1.2)		
Live-in treatment (non-hospital based)	5 (1)		
Other	3 (0.6)		
In-patient hospital mental health treatment program	2 (0.4)		
	**Satisfied n (%)**	**Dissatisfied n (%)**	
**Satisfaction with service type received** ^b^			
Sessions with private therapist/counsellor	34 (69.39)	15 (30.61)	
Mental health treatment by family physician or walk-in doctor	24 (68.57)	11 (31.43)	
Mental health supports with focus on education/employment goals	15 (65.22)	8 (34.78)	
Treatment by a psychiatrist	10 (66.67)	5 (33.33)	
Addiction assessment and/or treatment	7 (87.50)	1 (12.50)	
Supports for caregivers of someone with MHA concern	7 (77.78)	2 (22.22)	
Crisis supports/intervention	7 (77.78)	2 (22.22)	
Culturally specific mental health services	3 (50)	3 (50)	
Assessment by a psychiatrist	3 (37.5)	5 (62.5)	
In-patient hospital mental health treatment program	1 (50)	1 (50)	
Other	1 (33.33)	2 (66.67)	
Live-in treatment (non-hospital based)	1 (20)	4 (80)	
	**Yes n (%)**	**No n (%)**	
**Seeking to receive any of the following service types** ^b^			
Sessions with private therapist/counsellor	14 (28.57)	35 (71.43)	
Mental health treatment by family physician or walk-in doctor	13 (37.14)	22 (62.86)	
Mental health supports with focus on education/employment goals	7 (30.43)	16 (69.57)	
Treatment by a psychiatrist	4 (26.67)	11 (73.33)	
Culturally specific mental health services	3 (50)	3 (50)	
Other	2 (66.67)	1 (33.33)	
Live-in treatment (non-hospital based)	2 (40)	3 (60)	
Addiction assessment and/or treatment	2 (25)	6 (75)	
Crisis supports/intervention	2 (22.22)	7 (77.78)	
Assessment by a psychiatrist	1 (12.5)	7 (87.5)	
Supports for caregivers of someone with MHA concern	1 (11.11)	8 (88.89)	
In-patient hospital mental health treatment program	0	2 (100)	
	**Yes n (%)**	**No n (%)**	
**Thought about seeking but did not** ^a^			
None of the above	386 (77.2)	114 (22.8)	
Sessions with private therapist/counsellor	49 (9.8)	451 (90.2)	
Mental health treatment by family physician or walk-in doctor	35 (7)	465 (93)	
Mental health supports with focus on education/employment goals	27 (5.4)	473 (94.6)	
Treatment by a psychiatrist	19 (3.8)	481 (96.2)	
Supports for caregivers of someone with MHA concern	17 (3.4)	483 (96.6)	
Assessment by a psychiatrist	14 (2.8)	486 (97.2)	
Crisis supports/intervention	12 (2.4)	488 (97.6)	
In-patient hospital mental health treatment program	10 (2)	490 (98)	
Addiction assessment and/or treatment	7 (1.4)	493 (98.6)	
Culturally specific mental health services	6 (1.2)	494 (98.8)	
Live-in treatment (non-hospital based)	4 (0.8)	496 (99.2)	
Other	1 (0.2)	499 (99.8)	
	**n (%)**		
**Reason for not accessing service** ^b^			
Cost too much	28 (51.85)		
Not ready to connect with a service	21 (38.89)		
Not available in my preferred format	12 (23)		
Not available close to home	7 (12.96)		
Could not find desired service type	5 (9.26)		
I got connected but was put on a waiting list	2 (3.70)		
Decided to stick with previously connected to service	1 (1.85)		
Inadequate internet service to participate in online care	1 (1.85)		
	**Very to extremely helpful n (%)**	**Slightly to moderately helpful n (%)**	**Not at all helpful n (%)**
**Perceived helpfulness of MHA service delivery type** ^b^			
Primary healthcare provider	89 (60.14)	42 (28.37)	17 (11.49)
Navigator	75 (50.67)	56 (37.84)	17 (11.49)
Case manager	66 (44.59)	63 (42.56)	19 (12.84)
Information and referral services	58 (39.19)	76 (51.35)	14 (9.46)
Peer worker	55 (37.16)	71 (47.97)	22 (14.86)
Multimedia resources	53 (35.81)	79 (53.38)	16 (10.81)
	**Very to extremely helpful n (%)**	**Slightly to moderately helpful n (%)**	**Not at all helpful n (%)**
**Perceived helpfulness of MHA service delivery format** ^a^			
In-person one-on-one	166 (33.2)	164 (32.8)	170 (34)
Video conferencing one-on-one	100 (20)	175 (35)	225 (45)
One-on-one phone	91 (18.2)	191 (38.2)	218 (43.6)
In-person group	79 (15.8)	175 (35)	249 (29.8)
Online video conferencing group	71 (14.2)	152 (30.4)	277 (55.4)
Email communication	51 (10.2)	183 (36.6)	266 (53.2)
Text messaging	51 (10.2)	159 (31.8)	290 (58)
Phone group	51 (10.2)	146 (29.2)	303(60.6)

^a^The entire sample responded (n = 500).

^b^A subset of the sample responded (n < 500) and given that participants were in some cases permitted to respond to multiple options within a single item, discrepancies in percentages may be observed across questionnaire items.

Greater proportions of those that had accessed culturally specific (yes: *n* = 3, 50%; no: *n* = 3, 50%) and “other/unspecified” service types (yes: *n* = 2, 66.67%; no: *n* = 1, 33.33%), were still seeking services of this nature, while this was not the case for all other service types. Nearly 23% of the sample (*n *= 114) had considered seeking MHA services but did not. This was observed most for sessions with a private therapist/counsellor (*n *= 49, 9.8%), treatment by a family physician/walk-in doctor (*n* = 35, 7%), and support with focus on education/employment goals (*n* = 23; 4.6%). The top reasons for participants not accessing desired services were service costs (*n* = 28, 51.85%), not feeling ready to connect with services (*n* = 21, 38.89%), and lack of service availability in preferred format (*n* = 12, 23%). Concerning perceived helpfulness of MHA service delivery types, primary healthcare workers (*n *= 89, 60.14%), navigators (*n* = 75, 50.67%), and case managers (*n *= 66, 44.59%) were most frequently rated as very to extremely helpful. One-on-one in-person (*n* = 166, 33.2%), video (*n* = 100, 20%), and phone (*n* = 91, 18.2%) delivery of MHA services were most frequently rated as very to extremely helpful, whereas group phone (*n* = 303, 60.6%), text (n = 290, 58%), and group video (*n* = 277, 55.4%) were least frequently rated as very to extremely helpful.

## 4. Discussion

### 4.1. Predictors of MHA concerns

Our findings suggest that, in our sample of northern Ontarians, the prevalence of MHA concerns may decrease slightly with increasing age. However, given that this effect was modest (OR = 0.972), it should be interpreted with caution. Similarly, a study assessing depressive symptoms across Canada found that advancing age was associated with fewer depressive symptoms in rural regions, though the effect was small [33]. Accordingly, further research is warranted to determine whether this effect would hold across other samples and contexts. Furthermore, middle/high SES, straight, unmarried, and participants satisfied with social support were less likely to screen positive for MHA concern (Table 2). However, a previous study found that being unattached, as opposed to married, is a risk factor for health concerns in northern Ontario [8]. Moreover, similar results are also found in samples from other geographic locations [34,35], suggesting that MHA concerns may be more common in unmarried individuals. One potential explanation for our contrary finding may relate to geographical barriers faced in northern Ontario. Fewer available general and specialized services and public transportation options contribute to the increase in unmet MHA needs [10,13]. Moreover, northern locales of Ontario have drastically lower population densities relative to other regions in the province [24]. These isolating conditions, coupled with potential effects of the COVID-19 pandemic, might mitigate the typically positive effects that being married has on mental health. Future work with larger samples may consider exploring potential interaction variables to help better understand this phenomenon.

### 4.2. MHA service-related experiences

Although 77% of the sample reported MHA concerns, only 23% of those with MHA concerns accessed support (Table 3). Moreover, an additional 23% of the sample thought about accessing MHA services but did not act on these thoughts (Table 4). Given that most of the sample reported MHA problems (meeting or exceeding threshold levels that signal for professional inquiry), it is concerning that less than a quarter of the individuals in our sample reporting MHA concerns had received support. Interestingly, this finding does not align with rates of service access in Ontario, in that estimates of Ontarians with MHA issues reporting that services did not meet their needs or that they did not access MHA services at all range from 8-33% [36,37]. Our estimate suggests that approximately 77% of residents screening for MHA concerns lack access to services, a stark contrast to overall estimates within Ontario. As our sample is comprised of northern Ontario residents, our findings may illustrate a more accurate, albeit unfortunate, representation of the current state of MHA care access in this region. Although, it is worth reiterating our employment of screening tools [29,30] (i.e., which indicate that if one exceeds a particular threshold, one may benefit from assessment) as opposed to diagnostic tools as indicators of MHA concern.

Among those who did access support, private therapy, caregiver supports, and support focusing on vocational goals were most accessed (Table 4). The most frequently reported services received were generalized (e.g., mental health treatment by family physician or walk-in doctor) rather than specialized (e.g., culturally specific mental health services); access patterns that have also been identified in previous work in other jurisdictions [23]. Service users tended to be more satisfied than dissatisfied with all services accessed, except for assessment by a psychiatrist, culturally specific supports, live-in supports, in-patient hospital treatment, and “other/unspecified” service types (Table 4). These sources of dissatisfaction may reflect the lack of available, community-tailored, and diverse service options. For example, northern Ontario youths with substance use concerns expressed that there were fewer specialized programming options available as support [19]. Additionally, insufficient recruitment and retention of physicians [38] and other MHA care providers [39] in northern Ontario might explain this relative lack of sufficient and appropriate local MHA supports.

The most frequently reported barriers to service access included service costs, not being ready to engage with supports, as well as unavailability of services in preferred formats (e.g., one-one-one delivery), close to home, and of the desired service type (Table 4). Such barriers, which are predominantly systemic in nature, illustrate the vast obstacles northern Ontarians face when considering their MHA needs and preferences. Furthermore, the reported access barriers highlight existing systemic factors engrained in the MHA healthcare system within the northern regions of the province. For example, costs associated with travel to and from MHA services in addition to lengthy travel distances are common barriers to accessing MHA care in rural Canada [40]. MHA-related stigma and lack of anonymity within rural regions [20] may contribute to feelings of hesitation to seek out support. On the contrary, one of the least frequently reported barriers was having inadequate internet service to receive online support, which may reflect that due to online survey administration, our sample already included people with internet access [26]. It may also reflect a shift to the use of more virtual MHA care options due to both preference and necessity.

Reported needs and preferences of our sample suggest that such barriers might be surmounted by incorporating services delivered by primary healthcare providers, navigators, and case managers. Moreover, increased access might be accomplished by offering one-on-one support with in-person, video, or phone call options (Table 4). Our findings align with and build on the existing literature that has investigated rural-specific solutions to MHA access barriers. There is widespread support for incorporating more virtual technologies to provide remote care [10,41] through telemedicine [24] or primary care providers with MHA specialty via phone or video call [39]. Needs may also be addressed by providing more accessible publicly funded services, especially those focusing on practical needs like housing, finances, and transportation [42]. Furthermore, appropriate care may be provided by developing and involving community-based MHA programs that consider and align with local community members’ needs, preferences, and values [43,44].

### 4.3. Study limitations

One limitation of the current study is that data were collected during tail end of the peak of the COVID-19 pandemic. Consequently, our results may reflect MHA service experiences (i.e., accessed supports and barriers) as they occurred in the context of the COVID-19 pandemic and the subsequent physical, emotional and resource limitations [45]. However, given the COVID-19 pandemic “aftershocks” are expected to continue in the MHA sector for many years, the findings presented in the current study likely remain important for service planning and allocation. Another limitation of the current study involves disparities in racial representation within the sample. Indigenous individuals have been identified as at-risk for experiencing mental health concerns [8,25]. The same is observed for Black and other people of colour: Mental health concerns are especially high across Canada [46]. Although Indigenous identity was amongst the largest group of Black, Indigenous, and people of colour (BIPOC) individuals in our sample, BIPOC participants were grouped in analysis due to sample size. As a result, predictors of MHA concerns for BIPOC participants, specifically, as well as their care experiences, are not adequately distinguished in this study. Future work should include sufficient numbers of people from equity-deserving groups in northern, rural, and remote regions to better understand their unique and specific MHA needs.

Additionally, given that our survey was only administered online, responses were limited to northern Ontario residents with internet access. This prevented us from collecting data from those without internet access, a limitation that may be addressed by distributing surveys via mail or in person. Along similar lines, the present research collected online self-report data, which may be methodologically less ideal than objective clinical assessment. Despite this potential limitation, research suggests that online collection of self-reported psychiatric symptoms is a fair, and relatively accurate way to gauge psychopathology, particularly when accompanied by other methods, for example, in-depth interviews [47].

Furthermore, despite collection of a geographic variable (first three digits of postal code) and access to a sample of Ontario residents living outside of northern Ontario [26], we did not include a geographic variable in our adjusted logistic regression model (i.e., differentiating specific regions within northern Ontario) or conduct any comparison analyses between northern Ontario and other regions (eastern, southern, and western Ontario). Given the current study’s overarching goal of exploring predictors of MHA concern and experiences in accessing care specifically in northern Ontario, we focused on this specific regional context, as opposed to drawing comparisons between locales both within and outside of northern Ontario. Although region may act as an unobserved confounder in the present study, with established knowledge of the greater MHA support disparities experienced in northern Ontario relative to other Ontario locales [15], our focus was to highlight potential contributors to such disparities, while also providing respondents with a platform to express their service-related needs. Finally, this study was cross-sectional in nature and non-probabilistic sampling methods were used. This field of research might be enhanced by a more longitudinal exploration of how needs and service utilization change over time. Probabilistic sampling may also be used to ensure all northern Ontario residents have an equal likelihood of being sampled.

### 4.4. Implications and conclusions

The current study presents details of predictors, experiences, and needs of northern Ontario residents navigating MHA concerns. Accordingly, we found that sociodemographic characteristics, namely age, SES, sexual orientation, marital status, and satisfaction with social support independently predicted presence of MHA concerns. Other variables such as gender, education level, race, and neutral satisfaction with social support did not independently predict the presence of MHA concerns. Combined with reported experiences of MHA service access and needs, these findings can inform future efforts to resolve MHA care inequities in northern Ontario and other remote and rural regions. For example, participants’ desire for more individual in-person support and access to primary healthcare providers may drive government initiatives to recruit, train, and ultimately retain more physicians and MHA care providers in these regions [48,49]. As cost was the most frequently rated barrier to accessing MHA services, increased investment in publicly available MHA services, particularly in remote and rural regions, should also be prioritized. Accordingly, results from the current study should be considered when developing and implementing locally tailored MHA supports.
